# Clinical Protocol Effects With LED Photobiomodulation for Reducing Adipose Tissue in the Abdomen Region

**DOI:** 10.1111/jocd.16675

**Published:** 2024-12-08

**Authors:** Michele Akemi Nishioka, Patricia Brassolatti, Ana Carolina Araruna Alves, Jéssica Helena Franco Dorigatti, Fabiele Chieregato, Stephani de Almeida, Stephany Luanna Queiroga Farias, Patricia Froes Meyer, José Ricardo de Souza

**Affiliations:** ^1^ Department of Research Development and Innovation at Brazilian Medical Equipment Industry (IBRAMED) Amparo Brazil; ^2^ Federal University of Rio Grande do Norte (UFRN) Natal Brazil; ^3^ Brazilian Medical Equipment Industry (IBRAMED) Amparo Brazil

**Keywords:** adipose tissue, body measurement reduction, LED phototherapy, lipolysis, red and infrared

## Abstract

**Background:**

Reverse the fat accumulation and to improve body contouring with more safely than invasive procedures has become a necessary therapeutic approach, but little detailed when using LED photobiomodulation.

**Aims:**

To evaluate an application protocol with two different wavelengths (red and infrared LED) consecutively in the abdomen region alone or associated with the dermocosmetics application with lipolytic properties.

**Methods:**

Ninety patients with a significant amount of adipose tissue in the abdomen region were selected and randomized into three groups: Sham (SG), in which patients received a simulation of the treatment; LED (LG), in which participants received the application with LED; LED with dermocosmetic (LDG), in which the participants underwent a combination of LED and dermocosmetic treatment. The following assessments were carried out: anthropometric data (weight, height, perimetry, and adipometry); ultrasound examination to adipose layer; patient satisfaction questionnaire; histology of a donated a tissue sample.

**Results:**

The main findings were a decrease in umbilical perimetry, a significant decrease in the body fat layer determined by ultrasound and significant histological changes that indicated an improvement in the appearance of the skin and an increase in the amount of macrophages in the subcutaneous layer. Histological data also showed an improvement in the appearance of the skin with an increase in collagen deposition and an increase in macrophages in the subcutaneous layer.

**Conclusions:**

The LED phototherapy application protocol, both alone and in association with the dermocosmetic, with the emission of two consecutive wavelengths, was effective in reducing the fat tissue in the abdomen region.

## 1 Introduction

The adipose tissue has the auxiliary function of the thermal maintenance of body structures and is still capable of storing significant amounts of energy reserves [1]. Generally, when there is a greater amount of energy consumed than the body's need for metabolic expenditure, this energy is accumulated in the form of fat, present inside the adipocytes [2]. This fat accumulation may also be associated with gender, age, metabolic and genetic conditions, environmental and nutritional factors [2, 3]. One of the alternatives to help reverse fat accumulation consists of treatments with non‐invasive techniques, which have been investigated due to ease of application, low risk compared to major surgeries and faster recovery time.

Among non‐invasive techniques, technologies that use light to stimulate the cellular environment stand out [4, 5, 6, 7]. The physiological effects by photobiomodulation therapy (PBMT) in the treatment of localized fat relate to the activation of the mitochondrial photoreceptor cytochrome c oxidase, which stimulation of cellular metabolism by increasing the trifosfato de adenosine (ATP) [8, 9]. In addition, this biochemical activity also induces the upregulation of cAMP, which stimulates cytoplasmic lipase and converts triglycerides into fatty acids and glycerol, becoming more available for metabolism [8]. Yet, the lipolysis mechanism resulting from PBMT may also be related to the increase in oxidative stress via reactive oxygen species (ROS) that can cause microdamage to lipids and lead the apoptosis cells [8, 9, 10].

Then, given the physiological effects of PBMT against the adipocyte layer, it becomes promising to use light‐emitting diodes (LED) to improve body contouring with more safely than invasive surgical procedures. There is evidence that shows a significant reduction in abdominal circumference after application of the red laser [11, 12], however, the literature lacks reports of the LED's effect on body reduction, either applied alone or in combination with a dermocosmetic with specific properties to reduce body size. Therefore, the aim of this study was to evaluate the effect of LED PBMT, associated or not with a thermogenic dermocosmetic, in reducing localized abdominal fat and improving body contour.

## 2 Materials And Methods

### 2.1 Study Design and Subject Population

The study was approved by the ethics and research committee (number 3913119) and was developed in a physiotherapy clinic located in the city of Natal‐RN/Brazil. Ninety women aged between 30 and 45 years, sedentary, with a body mass index (BMI) considered between normal and overweight (18.5–29.9) and skin folds above 1.5 cm, were recruited. Participants were randomized into three distinct groups with 30 individuals in each group, as described below:

- Sham group (SG): Participants underwent LED PBMT treatment, but with the equipment turned off;
- Group treated with LED (LG): Participants underwent with LED PBMT at wavelengths of 630 and 850 nm with the parameters described in Table 1;
- Group treated with LED associated with dermocosmetic (LDG): Participants underwent with LED PBMT at wavelengths of 630 and 850 nm with the parameters described in Table 1, associated with the use of dermocosmetic.

**TABLE 1:** Irradiation parameters of the LG and LDG groups.

	Red LED	Infrared LED
Wavelength (nm)	630	850
Spot area (cm^2^)	80	80
Power (mW)	2700	3150
Irradiance (W/cm^2^)	0.033	0.039
Total energy (J)	416	267
Fluence (J/cm^2^)	6	4
Irradiation time (s)	177 s	101 s
Number of applied points	4	4
Applied area (cm^2^)	400	400
Number of treatment sessions	2 times per week	2 times per week

The study participants were first submitted to anthropometric data collection (weight, height, perimetry, and adipometry), and ultrasound examination to assess adipose layer. The assessments took place at three different times: before starting treatment, after five sessions and at the end of the treatment, after 10 sessions. In the end of the 10 sessions, all participants also answered the patient satisfaction questionnaire.

In addition, to evaluate the histological conditions of the tissue, one participant from each group had an adipose tissue sample collected for histological analysis. Furthermore, all treatment interventions were performed by the same therapist and data collection and analysis were performed by a blind evaluator.

### 2.2 Photobiomodulation Therapy

The Sham and active PBMT groups were performed using the Antares equipment, manufactured by IBRAMED—Brazilian Medical Device Company. Participants in the LG and LDG groups were irradiated using the cluster device of Antares (IBRAMED, Amparo, SP, Brazil), initially applying the red LED (630 nm) and immediately followed by the near infrared LED (850 nm), with the parameters described in Table 1. The irradiations were carried out twice a week, with a minimum interval of 48 h between sessions, until completing 10 treatment sessions. Four sites were defined, distributed in two applications in the lower abdomen and two in the upper abdomen, with the left side treated and the right side considered as control.

Before the intervention, an appropriate asepsis of the irradiated area with 70% alcohol was performed, regardless of the group to which the participant was allocated. All the groups always started with the LED PBMT application. In the LDG participants, a more intense cleaning was also carried out with prior exfoliation of the area to be treated as described in the session referring to the application of the dermocosmetic.

### 2.3 Dermocosmetic Application

Before starting the treatment, the LDG participants underwent an allergy test, in which a small portion of the product was applied to a tissue area to observe possible changes and/or irritability. After confirming that there were no risks of allergy regarding any of the components, the gel was applied at the time of the session always after the application of the PBMT.

After LED PBMT application, a new cleaning the area treated was done with a cotton pad soaked in water and liquid soap ( *Aloe vera* 2% and Hamamelis 1%) in circular movements. Afterward, the tissue was exfoliated with a gel with 3% green microspheres. Afterward, the tissue was exfoliated with a gel with 3% green microspheres and a dermocosmetic with measurement reduction properties containing lypoxyn 5%, liporeductyl 5%, coheliss 4%, grape seed oil, 3% cream qsp was applied.

### 2.4 Data Collect

#### 2.4.1 Anthropometric Data

For the data weight and height, was used a mechanical stadiometer (model 110 CH; Welmy, SP, Brazil) and then the BMI calculation (kg/height m^2^) was performed. For the perimetry, was measured using a tape measure (RMC or Fiber). This measurement was performed on the abdominal circumference respecting the previously determined horizontal lines located in the lower and upper abdomen.

For the adipometry, was used with the equipment (CESCORF or Sanny). The alignment was standardized for all evaluations to two centimeters from the umbilicus, parallel to the longitudinal axis. So, two determined skinfolds, one 5 cm above and one 5 cm below the umbilical line, both in the right and left abdomen, using the marking of the first application point as a reference. The assessment was performed on both the right and left sides of the abdomen. Three consecutive measurements were obtained, and then, an average was assigned as the final value, thus determining the size of the fat fold.

#### 2.4.2 Adipose Layer Evaluation by Imaging Ultrasound

The patients were positioned in dorsal decubitus for the evaluation of the diagnostic ultrasound in the abdomen region, which was performed using the punctual technique. The demarcation of the evaluation points was carried out 5 cm above or below the umbilical line and 5 cm demarcated toward the right or left abdomen, thus determining the evaluation points for the supraumbilical and infraumbilical regions. For this evaluation, a linear transducer was used (frequency 6–18 MHz) (MYLAB 25 GOLD, ESAOTE, ITALY) with the aid of the VPAN software (ESAOTE, ITALY) for the construction of panoramic images. The examination and analysis of the images were performed by an experienced specialist physician.

#### 2.4.3 Patient Satisfaction Questionnaire

At the end of the treatment, the participants answered the Satisfaction Questionnaire Segot‐chicq et al. [13] adapted, which is used to classify the participant's perception of the treatment.

#### 2.4.4 Histology

The adipose tissue samples provided were fixed in 10% buffered formalin (Merck, Darmstadt, Germany), embedded in paraffin and transversal cut to 5 μm thickness. Three sections were obtained from each sample, which were stained with hematoxylin and eosin (HE, Merck). Histological analyses were carried out with the aid of a light microscope (Zeiss Axioshop, Carl Zeiss, Rio de Janeiro, Brazil), with a 40× objective and by a blind evaluator to the study groups [14]. The parameters evaluated qualitatively were collagen fibers thickness, presence of fibroblasts, and cells inflammatory.

#### 2.4.5 Immunohistochemistry

Histological specimens 4 μm thick were placed on silanized slides and kept in an oven at 37°C for 24 h. After this period, histological sections were deparaffinized, hydrated, marked with a hydrophobic pen, and washed twice in a Tween enriched buffer solution for 3 min. In sequence, the sections were immersed in a hydrogen peroxide for 10 min, washed twice in phosphate‐buffered solution (PBS) for 3 min, and immersed in inactivate endogenous peroxidase for 30 min [14].

So, the samples were incubated with the anti‐CD68^+^ antibody (Creative Biolabs) at a 1:200 concentration for 2 h, washed three times in PBS, and was applied of the avidin‐biotin complex (Vector Laboratories) for 45 min. For the visualization of the complexes, 0.05% 3′3‐diaminobenzidine solution was applied, and Harris Hematoxylin (Vector Laboratories) was used for contrast [14]. The qualitative analysis was taken by a blind evaluator to the study groups, using a light microscope (Zeiss Axioshop, Carl Zeiss, Rio de Janeiro, Brazil) with a 40x objective. The qualitative analysis was taken by a blind evaluator to the study groups, using a light microscope (Zeiss Axioshop, Carl Zeiss, Rio de Janeiro, Brazil) with a 40× objective, to identify the macrophages presence.

### 2.5 Statistical Analysis

The data passed the Levene and Shapiro–Wilk tests and were analyzed using the two‐way ANOVA test with Tukey post hoc. All statistical analysis were performed using the GraphPad Prism software (version 9.4.1 for Windows), with a 5% level being considered a significant value.

## 3 Results

### 3.1 Anthropometric Data

Regarding body mass index and the average of the measurements performed with an adipometer, no statistically significant differences were observed between groups and periods evaluated.

In the perimetry evaluation, there was a reduction in abdominal circumference in the LG and LDG, with a statistical difference in relation to the SG (*p* = 0.02). Comparing the LG with the LDG, a statistically significant difference was also found, with reduction observed in the LG group (*p* = 0.01).

### 3.2 Imaging Ultrasound Assessment

**FIGURE 1:**
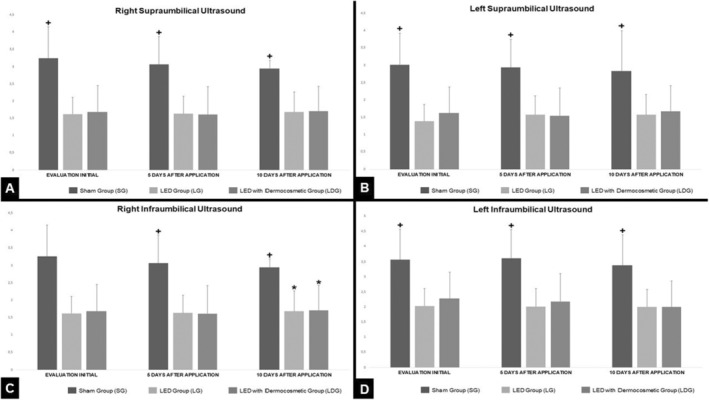
Adipose layer assessment by imaging ultrasound. (A) Right supraumbilical region; (B) left supraumbilical region; (C) right infraumbilical region; (D) left infraumbilical region; (+) differences between the sham group; (*) differences between treated groups.

In the evaluation of the right supraumbilical region, there was significant reduction in the adipose layer of the LG and LDG in relation to the SG, however without any statistical difference determined in the comparison of LG with LDG (Figure 1A). The left supraumbilical region showed similar results, with a statistically significant difference only when comparing the groups treated with the SG (Figure 1B).

In the evaluation of the right infraumbilical region, it was possible to identify a statistically significant difference in relation to the decrease in the adipose layer in the LG and LDG when compared to the SG. In addition, a statistically significant difference was also presented between LG and LDG groups, in which LG showed a significant reduction (Figure 1C). The left infraumbilical region showed similar results, with a statistically significant difference when the LG and LDG were compared to the SG, but with no difference when comparing only the LG with the LDG (Figure 1D).

### 3.3 Satisfaction Rate

By evaluating the participant's reports regarding the treatment through a questionnaire, a high degree of satisfaction was obtained, quantitatively reported in 88% for the SG, 91% reported by the LG and 92% reported by the LDG.

### 3.4 Histological Analysis

#### 3.4.1 Collagen and Fibroblasts

**FIGURE 2:**
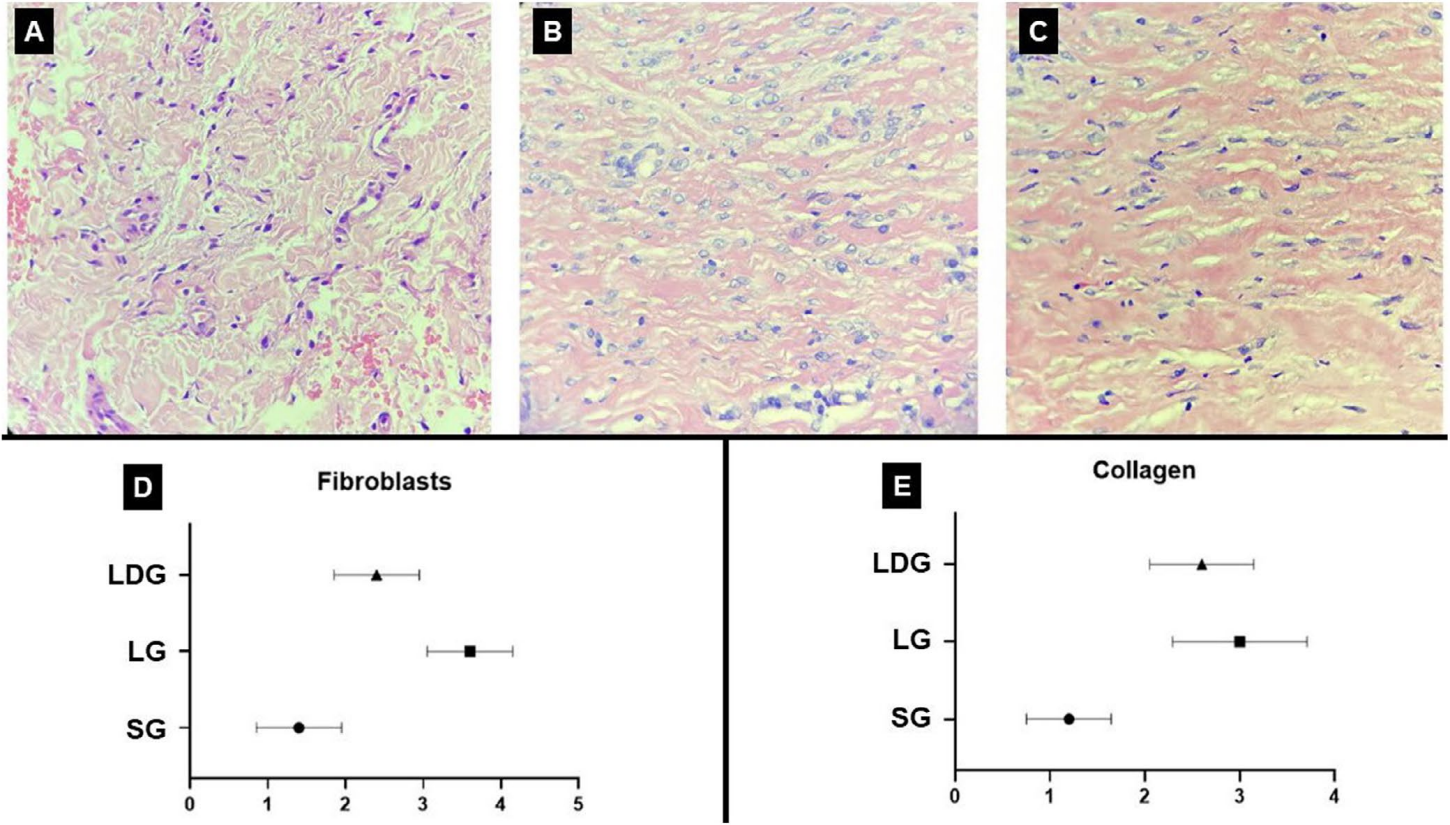
Representative photomicrographs of tissue stained with HE and statistic graphs representatives. (A) Sham group (SG); (B) LED group (LG); (C) LED and dermocosmetic group (LDG); (D) statistic graph about the amount of fibroblasts; (E) statistic graph about the collagen evaluation.

In the histological evaluation, it was possible to observe that the SG presented collagen fibers in normal thickness with the presence of inactive and sparse fibroblasts (Figure 2A). The LG presented the presence of fibroblasts with hyperplasia, demonstrated by the increase of cytoplasm and cellular nuclei, and intense deposition of collagen (Figure 2B). The LDG showed an increase in the amount of fibroblasts with collagen deposition (Figure 2C), however more discreet than that founding in the LG.

Statistically, it was possible to identify a difference in the amount of fibroblasts when comparing the SG to the LG, in which the LG showed the highest number of cells (Figure 2D). Regarding the collagen evaluation, a statistically significant increase was observed in the LG when compared to the SG; however, no statistically significant difference was found when comparing the LG to the LDG (Figure 2E).

#### 3.4.2 Adipose Cells and Inflammatory Response

**FIGURE 3:**
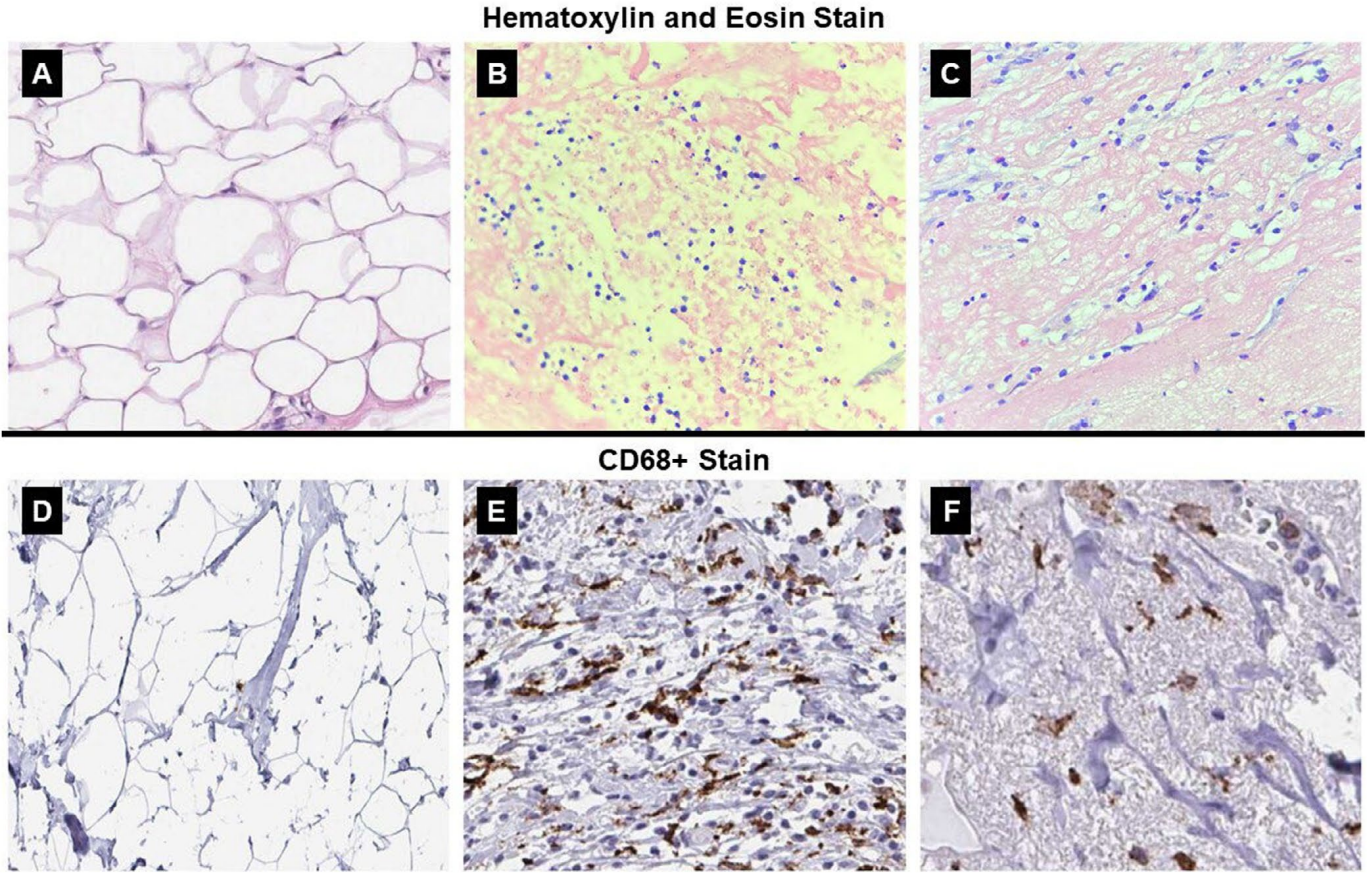
Representative photomicrographs of the adipose tissue stained with HE and stained with CD68^+^. (A) Adipose cells of the sham group; (B) adipose cells of the LED group; (C) adipose cells of the LED with dermocosmetic group; (D) absence of CD68^+^ macrophages in the adipose cells of the sham group; (E) expressive presence of CD68^+^ macrophages in the adipose cells of the LED group; (F) moderate presence of CD68^+^ macrophages in the adipose cells of the LED with dermocosmetic group.

In the SG, adipose tissue with normal characteristics was observed (Figure 3A) and the absence of CD68^+^ macrophages (Figure 3D). In the LG, an indication of a process of necrosis of the adipose tissue was observed with the presence of cells with inflammatory characteristics (Figure 3B) and expressive presence of CD68^+^ macrophages (Figure 3E). In the LDG cells with inflammatory characteristics were also observed (Figure 3C) and a moderate amount of macrophages marked with CD68^+^ (Figure 3F).

**FIGURE 4:**
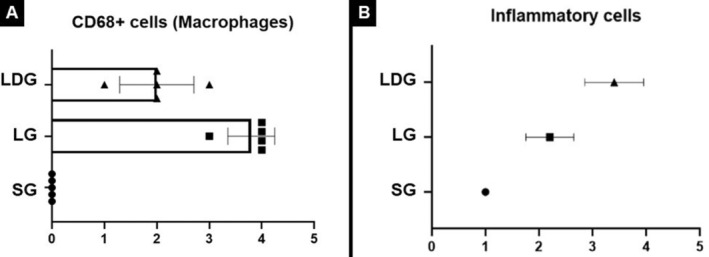
CD68^+^ stain and statistic evaluation of inflammatory cells (A). LDG, LED and dermocosmetic group; (B) LG, LED group; (C) SG, Sham group.

Statistically, the macrophages CD68^+^ was increased significantly in the LG when compared to the other evaluated groups. Regarding LDG, despite demonstrating marking referring to the presence of macrophages CD68^+^, the amount identified was lower than LG and higher than SG (Figure 4A). About the evaluation cells inflammatory, the LDG presented an increase significant when compared to SG (Figure 4B).

## 4 Discussion

Currently, the literature demonstrates scientific evidence related to the ability of PBMT to reduce subcutaneous adipose tissue and consequently improve abdominal circumference [5, 11, 12, 15]. The mechanisms involved cite mitochondrial action via an increase of ROS, which induces the formation of transient pores in the adipocyte membrane and thus allows the intracellular release of lipid content [10], as well as the activation of protein kinase, responsible for the induction of cytoplasmic lipase [8, 9], which also makes the fat content available to be subsequently catabolized by the body.

In view of this, the results of our study indicate the change in cellular bioenergetic state through the significant inflammatory process with a consequent increase in the macrophages number identified in the CD68^+^ staining. This marking refers to a possible phagocytosis process, which in this case can be directed to adipocytes and may indicative of beginning lipolysis process [16]. In the histological analysis, there was an increase in the number of fibroblasts with significant collagen deposition for groups treated with LED, but in the group with the dermocosmetic combination, this modification was more discreet when compared to the one that received only the LED. This increasing can be explained by the direct action of light, which activates the electron transport chain via cytochrome C oxidase and increases cell proliferation [9].

For more, our study identified reduction in the adipose layer detected in the evaluation of perimetry and ultrasound analysis. Jackson et al. [5] described the effects of red laser treatment in 632 patients who received six sessions directed at the waist, hip, and inner thigh regions. The authors pointed out that there was a reduction in abdominal circumference and which the sum of the reduction of all treated regions showed a statistically significant. This was allowed that the authors to conclude that laser light therapy is safe and effective for treating body fat. Corroborating our results regarding the application of light to adipose tissue, the study by Elnaggar [17] indicates that phototherapy was more effective in reducing the waist/hip ratio, followed by a reduction in the abdominal fat layer.

Adatto et al. [18] also highlighted in their study that the association of different therapies, including the use of laser light, is effective in reducing the volume of adipose tissue and skin sagging. As well as Mostafa and Elshafey [19], who compared the effects of laser versus cryolipolysis on abdominal adiposity and observed the same effects in relation to the reduction of local fat content. In the same sense, the study by Nestor et al. [20] demonstrated a significant reduction in arm circumference after six treatment sessions with the red laser only, which proves the effects of light specifically on adipocytes.

However, despite the various reports in the literature regarding the interaction of light with adipose tissue, most authors point to the use of laser, with the LED effects for this therapeutic purpose still being little explored. Yoon and Kim [21] address the use of LED and report their benefits when applied to adipose tissue in an experimental model. The authors observed that irradiation at a depth of 4–5 cm through the skin can reach fat cells and promoting a reduction in their internal content. Yanina et al. [22] evaluated the LED actions also in an experimental model and observed that light has an optimal penetration depth in the fat of rats of 2.49 mm when using a 625‐nm wavelength. Furthermore, the authors point out that the LED has advantages because it is low cost and durable, being able to reduce the impedance presented by the adipose tissue and thus achieving good interaction with these cells.

Another treatment approach that we also evaluated was to associate LED with a dermocosmetic with properties for lipolytic activity and blood microcirculation activation, with principles capable of stimulating TNF‐alpha and increasing cyclic‐AMP, which theoretically facilitates the release of the triglyceride present within the adipocyte. This approach was chosen to investigate whether this association would intensify the reduction of abdominal fat. However, our results did not indicate greater fat reduction with the combination treatment. There are authors point out that the dermocosmetics use with active principles intended for the lipolysis process are effective and present good results [23, 24]. But it should be noted that there are different formulations, and the diversity in the active principles association can provide different results.

In view of the findings of the present study, it is possible to define that the use of phototherapy with LED light is capable of interacting with adipocytes, causing interesting changes that may result in a decrease in their internal content related to triglycerides. In the case of association with the dermocosmetic, the product did not intensify the lipolysis action in adipocytes. Thus, it is possible to highlight that LED PBMT with protocol that uses two different wavelengths, red and infrared simultaneously, was effective and safe to mobilizing the fat content present inside the adipocytes in the abdomen region, providing a reduction in measurements and improved in body contour.

## Author Contribitions

**Michele Akemi Nishioka**, **Ana Carolina Araruna Alves**, **and Jéssica Helena Franco Dorigatti:** conception, design, and revision critical of the intellectual content. **Patricia Brassolatti:** conception, design, drafting, and revision critical of the intellectual content. **Fabiele Chieregato and Stephani de Almeida:** acquisition and interpretation of the data. **Patricia Froes Meyer:** revision critical of the intellectual content. **José Ricardo de Souza:** conception and revision critical of the intellectual content. **Stephany Luanna Queiroga Farias:** data analysis.

## Conflicts of Interest

The authors declare no conflicts of interest.

## Data Availability

Research data are not shared.
